# Strategies to improve PrEP uptake among West African men who have sex with men: a multi-country qualitative study

**DOI:** 10.3389/fpubh.2023.1165327

**Published:** 2023-04-25

**Authors:** Thijs Reyniers, Stéphane Alain Yoro Babo, Mamadou Ouedraogo, Ibrahima Kanta, Laurette Ekon Agbégnigan, Daniela Rojas, August Eubanks, Camille Anoma, Ter Tiero Elias Dah, Ephrem Mensah, Bintou Dembélé Keita, Bruno Spire, Bea Vuylsteke, Christian Laurent

**Affiliations:** ^1^Department of Public Health, Institute of Tropical Medicine, Antwerp, Belgium; ^2^Espace Confiance, Abidjan, Côte d'Ivoire; ^3^Association African Solidarité, Ouagadougou, Burkina Faso; ^4^ARCAD Santé Plus, Bamako, Mali; ^5^Espoir Vie Togo, Lomé, Togo; ^6^Aix Marseille Univ, INSERM, IRD, SESSTIM Sciences Economiques & Sociales de la Santé & Traitement de l'Information Médicale, Marseille, France; ^7^Coalition Plus, Community-based Research Laboratory, Pantin, France; ^8^UFR Sciences de la Santé, Université de Ouahigouya, Ouahigouya, Burkina Faso; ^9^TransVIHMI, Univ Montpellier, IRD, INSERM, Montpellier, France

**Keywords:** HIV prevention, PrEP, MSM, West Africa, health behavior, HIV, stigma

## Abstract

**Introduction:**

West African men who have sex with men (MSM) remain at substantial risk of contracting HIV. Pre-exposure prophylaxis (PrEP) can be an effective game-changer in reducing the number of HIV infections in MSM communities. To optimize the roll-out of PrEP, we need to better understand how we can increase its uptake. The objective of this study was to explore the perceptions of West African MSM toward PrEP and their proposed strategies to overcome barriers to PrEP uptake within their communities.

**Methods:**

Between April 2019 and November 2021, we conducted 12 focus group discussions with 97 MSM not taking PrEP, and 64 semi-structured interviews with MSM taking PrEP, in Burkina Faso, Côte d'Ivoire, Mali, and Togo. Data collection and analysis were guided and conducted by local research teams, enabling a community-based participatory approach. A coordinating researcher collaborated with these local teams to analyze the data guided by a grounded theory approach.

**Results:**

The results show that participants were generally positive toward PrEP and that MSM communities have become more aware of PrEP for the study. We identified three main strategies for increasing PrEP uptake. First, participants proposed to raise awareness and improve knowledge of HIV as they considered the self-perceived risk of MSM in their communities to be low. Second, because of existing misconceptions and false information, participants proposed to improve the dissemination of PrEP to allow for informed choices, e.g., via peers or PrEP users themselves. Third, as oral PrEP also entails a risk of being associated with HIV or homosexuality, strategies to avoid stigmatization (e.g., hiding pills) were deemed important.

**Discussion:**

These findings indicate that the roll-out of oral PrEP and other future PrEP modalities should be accompanied by raising awareness and improving knowledge on HIV, and wide dissemination of information that focuses on the health-promoting aspect of these tools. Tailored delivery and long-acting PrEP modalities will be important to avoid potential stigmatization. Sustained efforts to prevent discrimination and stigmatization based on HIV status or sexual orientation continue to be highly important strategies to address the HIV epidemic in West Africa.

## 1. Introduction

Men who have sex with men (MSM) are at substantial risk of HIV infection globally, and in particular, in sub-Saharan Africa (1). In West and Central Africa, the estimated HIV prevalence of 7.1% among MSM and 21.9% among transgender persons is much higher than in the general population (i.e., 1.3%) (2). Aside from biological factors (e.g., a higher risk per act for receptive anal sex) and sexual behavior factors (e.g., a higher average number of sexual partners), the increased risk of HIV acquisition may be explained by a social context that is less favorable for MSM (3). As a result, the organization of HIV prevention services faces additional difficulties due to stigmatization, discrimination, and even criminalization of homosexuality (e.g., in Togo) (2, 4–7). Therefore, strategies to strengthen HIV prevention efforts among West African MSM continue to be badly needed.

The preventive use of oral antiretroviral HIV medication, i.e., pre-exposure prophylaxis (PrEP) is a very efficacious HIV prevention tool if correctly taken (8, 9). By the third quarter of 2022, at least 3.3 million persons had initiated PrEP worldwide, the majority in high-income countries and Eastern and Southern Africa (2, 10). If we want to stop AIDS as a public health threat by 2030, UNAIDS calls for ensuring availability of PrEP for at least 10 million people at substantial risk of HIV by 2025 (2). To achieve such an ambitious target, we require more insights into the scaling up of efficacious HIV prevention tools within hard-to-reach and vulnerable communities such as MSM in West Africa. Hence, it is crucial to understand to what extent they consider PrEP an acceptable HIV prevention method and what may influence its uptake (11). Taking into account studies conducted between 2009 and 2016, the global acceptance of using PrEP among MSM is estimated to be 57.8% (12). Among MSM from low- and middle-income countries, the willingness to take PrEP ranges from 19.1 to 96.2% (13). It remains unclear to what extent such large differences in willingness to take PrEP are related to different social contexts, the period in which such studies were conducted (i.e., acceptability may have increased over time), or methodology (e.g., which samples were taken and how). To understand how PrEP uptake among West African MSM could be improved in their respective social contexts, we require more in-depth insights into their experiences, perceptions, and attitudes in this regard.

Studies undertaken in Nigeria, Burkina Faso, Mali, Côte d'Ivoire, and Togo suggest that ~20–30% of MSM do not use a condom during anal intercourse (14–18). Another study among 564 West African MSM found that ~54% did not use condoms during anal sex consistently in the previous 6 months (19). Particular MSM sub-groups are more likely to engage in sexual activities associated with higher risk-taking, such as sex combined with drug or alcohol use, or transactional sex (17, 20). For such MSM in West Africa, PrEP can be a game-changer, particularly because it can be taken irrespective of the situation in which sex occurs (21). However, a social context in which homosexuality is stigmatized or even criminalized can operate as an important barrier to PrEP uptake. In West Africa, organizations promoting sexual and gender diversity can face legal barriers to their operation, and the promotion of information that involves same-sex relationships can be prohibited (2, 22). Approximately 65% of MSM in West Africa have not disclosed their sexual orientation to any family member (23), which could be a barrier to coming forward for PrEP. To understand how PrEP uptake can be optimized among West African MSM, we need to better understand how to overcome such potential barriers.

A multi-country cohort study “CohMSM-PrEP” was undertaken from November 2017 to April 2020 among more than 600 MSM in Burkina Faso, Côte d'Ivoire, Mali, and Togo. Participants were recruited via four community-based clinics in Abidjan (Côte d'Ivoire), Bamako (Mali), Lomé (Togo), and Ouagadougou (Burkina Faso). Participants needed to be 18 years or older and at substantial risk of acquiring HIV. Participants could choose between daily or event-driven oral PrEP and switch regimens, discontinue, or restart during follow-up visits. During these 3-monthly visits, participants completed questionnaires, and clinical data were recorded. The results demonstrated that PrEP use was feasible and acceptable and led to a decrease in HIV incidence (2.3 per 100 person-years, compared to 10.0 before 2017) (24). At the time of this present study, PrEP was only available through the CohMSM-PrEP study in Burkina Faso, Côte d'Ivoire, Mali, and Togo (24). Currently, PrEP is being implemented in these countries. Understanding how PrEP uptake can be optimized among those at the highest risk is a crucial next step.

The objective of this study was to explore the perceptions of West African MSM toward PrEP and their proposed strategies to overcome barriers to PrEP uptake within their communities.

## 2. Materials and methods

The present study was conducted parallel to the CohMSM-PrEP study (24). We conducted 12 focus group discussions (FGDs) with MSM not taking PrEP to explore the variety of perceptions toward PrEP within these communities. We conducted 64 semi-structured interviews with MSM using PrEP via the CohMSM-PrEP study to explore their experiences with PrEP uptake and use.

The research team consisted of local researchers with a social science background (SBY, MO, IK, and LE), MSM community research assistants, an experienced coordinating social science researcher (TR), epidemiologists, and clinic heads. This enabled a community-based participatory approach throughout the study (25). During a 1-week workshop, we collaboratively decided upon participant selection criteria and developed the topic guides with the research teams of the four countries. The workshop also provided the opportunity to pilot the data collection tools and to strengthen the qualitative research capacities. Data collection and analysis were based on a grounded theory approach (26), meaning that the topic guides and data coding frameworks were optimized iteratively in close collaboration with the local social science researchers.

### 2.1. Focus group discussions with MSM not taking PrEP

We conducted the FGDs according to good and common practices (27). During the FGDs, participants were encouraged to share and compare their opinions and experiences, adopting an interactive approach to explore the variety of their perspectives toward PrEP (27).

We aimed to include 6–8 participants in each FGD but invited more to anticipate potential drop-out. In each country, we conducted three FGDs: two with HIV-negative MSM (25 years old or younger, and over 25 years old) and one with MSM living with HIV. Participants were recruited via peer educators from the CohMSM-PrEP study. To include a wide variety of perspectives and opinions, peer educators were encouraged to recruit participants who did not yet have direct linkages with the HIV clinic and to ensure sufficient variation in terms of sociodemographic background (e.g., the district where they lived).

The FGDs were moderated by an experienced local social science researcher, assisted by a peer educator or community research assistant. They were conducted in safe spaces, such as a meeting room within the clinic or a closed bar frequented by MSM. Before the FGD, participants needed to complete a brief questionnaire that inquired about their sociodemographics and PrEP awareness. After having been informed about the study and its procedures, participants were able to ask questions and were instructed to complete a consent form. The research assistant ensured that all forms were completed and helped participants when requested. The topic guide included open-ended questions to foster discussion on HIV, HIV prevention strategies, PrEP, barriers to PrEP uptake, and strategies to overcome such barriers (See Supplementary material for topic guide). Information on PrEP was introduced during the FGD so that participants could provide informed opinions. We used case vignettes (e.g., a case of “Jean” not taking PrEP despite being at risk of HIV acquisition) so that participants were not obliged to talk about their own experiences, considering the sensitivity of the subject. The FGDs were conducted in French or the local language. Participants received a transportation fee of ~€10.

### 2.2. Interviews with PrEP users

We considered interviews to be more appropriate to explore personal experiences with PrEP uptake and use (28). They were semi-structured, meaning that they included a basic set of questions but allowed for flexibility in the order and manner in which they were posed. Having a structure was considered important to allow for consistency between interviews and sites, while the flexibility allowed for a more natural flow of conversation.

Interviewees had to have been using PrEP for at least 2 months. The research team decided upon a broad range of selection criteria they considered important to ensure sufficient variation in opinions and experiences with PrEP use. At least two participants needed to be recruited for each of the following criteria: engaging in sex work, having multiple partners, having sex with women, having both insertive and receptive anal sex, changing condom use while on PrEP (both more or less), changing PrEP regimen (daily to event-driven or vice versa), adhering well, adhering poorly, or discontinuing PrEP. They were selected based on their answers in the questionnaires or the medical records of the CohMSM-PrEP study (24). They were recruited by the research community assistants.

The social science researcher in each country conducted 16 interviews, resulting in 64 interviews in total. They were conducted in the researcher's offices or in a safe space. For this analysis, we focused primarily on data pertaining to the process that resulted in uptake, i.e., their reactions the first time they heard of PrEP and the reasons why they started taking it. Interviews were either in French or local languages. Participants provided informed consent, completed an additional short questionnaire, and received a transportation fee of ~€10.

### 2.3. Data analysis

All audio recordings were transcribed verbatim and translated into French if necessary, by the local community research assistant. Transcripts were verified by the local social science researcher. Where possible, information that could directly identify the participant or any other person was pseudonymized during transcription. Data analysis was guided by a grounded theory approach (26), focusing on finding barriers to PrEP uptake and potential interventions to overcome such barriers. During the first round of analysis, the local social science researchers open-coded at least one-third of the transcripts, in collaboration with the coordinating researcher. An initial coding framework was decided upon and used to analyze subsequent transcripts collaboratively. The coordinating researcher re-analyzed all data to create an overarching coding framework. The final analysis and results were verified by the research team.

## 3. Results

### 3.1. Sample description

Between April 2019 and November 2021, we conducted 12 FGDs with 97 MSM in Burkina Faso (Ouagadougou), Côte d'Ivoire (Abidjan), Mali (Bamako), and Togo (Lomé). As shown in Table 1, ~51% of the FGD participants had completed or were completing higher education. Twenty-three (24%) were married or in a relationship. Approximately 43% considered themselves homosexual and 48% considered themselves bisexual.

**Table 1:** Focus group discussions with MSM not taking PrEP.

	**Country**	**Date FGD**	**Inclusion criteria**		**Participants**	**Highest educational level**		**Relationship status**	**Sexual orientation** ^**e**^		**Heard about PrEP^b^**
		**Month/year**	**HIV status**	**Age**	**Number**	**% Secondary**	**% Higher**	**% Single** ^a^	**% Homosexual**	**% Bisexual**	**% Yes**
1	Côte d'Ivoire	3/2020	HIV negative	<25 years	6	67%	33%	50%	17%	67%	83%
2	Côte d'Ivoire	4/2019	HIV negative	25+ years	10	60%	30%	60%	10%	90%	0%
3	Côte d'Ivoire^c^	8/2020	HIV positive	N/A	8	33%^c^	33%^c^	100%^c^	17%^c^	67%^c^	83%^c^
4	Mali	5/2019	HIV negative	<25 years	8	43%^d^	57%^d^	100%	75%	13%	38%
5	Mali	11/2019	HIV negative	25+ years	8	38%	63%	75%	38%	50%	50%
6	Mali	3/2021	HIV positive	N/A	6	50%	33%	83%	33%	50%	100%
7	Togo	5/2019	HIV negative	<25 years	11	37%	64%	36%	64%^e^	27%^e^	36%
8	Togo	7/2019	HIV negative	25+ years	8	25%	75%	75%	25%	38%	100%
9	Togo	10/2020	HIV positive	N/A	6	33%	67%	67%	83%	17%	100%
10	Burkina Faso	6/2019	HIV negative	<25 years	7	57%	29%	100%	29%^e^	57%^e^	43%
11	Burkina Faso	3/2021	HIV negative	25+ years	11	36%	55%	73%	27%^e^	55%^e^	91%
12	Burkina Faso	12/2020	HIV positive	N/A	8	38%	63%	100%	63%^e^	25%^e^	100%
				**Total**	97	43%^c, d^	51%^c, d^	77%^c^	43%^c, e^	48%^c, e^	65%^c^

FGD, focus group discussion; N/A, not applicable.

a Relationship status “single” includes “divorced,” “separated,” and “widow.”

b Proportion of FGD participants indicating they had heard about PrEP before participating in the FGDs.

c Missing questionnaire responses for two participants in FGD 3 (Côte d'Ivoire), not included in the totals.

d Missing answers to “Educational level” for one participant in FGD 4 (Mali), not included in the totals.

e Answers “preferred not to say” (n = 4): one in FGD 7, 10, 11, 12, not included in the totals.

We conducted 64 semi-structured interviews with PrEP users from the CohMSM-PrEP study. As shown in Table 2, the majority of the interviewees were younger than 30 years old (72%) and had completed or were completing higher education (59%). Fourteen (23%) were married or in a relationship. Approximately 45% of the interviewees considered themselves homosexual and 45% considered themselves bisexual. They had started taking PrEP between 2 and 48 months before the interview, with a median of 24 months.

**Table 2:** Interviews with MSM taking PrEP (*n* = 64, 16 per country).

**Country**	**Period of interviews**	**Age**	**Highest educational level**		**Relationship status**	**Sexual orientation**		**Months on PrEP**
	**M/Y-M/Y**	**%**<**30Y**	**% Secondary**	**% Higher**	**% Single** ^a^	**% Homosexual**	**% Bisexual**	**Median (min-max)**
Côte d'Ivoire^b^	4/2019–11/2021	92%^b^	9%^b, c^	91%^b, c^	100%^b^	67%^b^	33%^b^	18 (12–48)^b, d^
Mali	5/2019–10/2020	56%	31%	50%	81%	56%	31%	18 (9–36)^d^
Togo	05/2019–10/2020	69%	44%	56%	56%	31%	56%	24 (4–36)
Burkina Faso	6/2019–9/2021	75%	44%	50%	75%	31%	56%	31 (2–41)
	**Total**	72%^b^	34%^b, c^	59%^b, c^	78%^b^	45%^b^	45%^b^	24 (2–48)^b, d^

M, month; Y, year; min, minimum; max, maximum.

a Relationship status “single” includes “divorced,” “separated,” and “widow.”

b Missing answers for four participants from Côte d'Ivoire, not included in the totals.

c Missing answers for one participant from Côte d'Ivoire on “Educational level,” not included in the totals.

d Missing answers for one participant from Mali and one participant from Côte d'Ivoire on “Months of PrEP,” not included in the totals.

Below, we first describe their awareness and attitudes toward PrEP, followed by three proposed strategies for increasing PrEP uptake: increase awareness and improve knowledge of HIV and HIV risk (i); improve the dissemination of PrEP information to allow for informed choices (ii); and implement strategies to avoid PrEP stigma (iii).

### 3.2. PrEP awareness and attitudes

#### 3.2.1. Awareness of oral PrEP

In general, the FGD participants were very curious about PrEP, as they had never used it before. The questionnaires revealed that ~28% (10/36) of the participants in the first FGD of each country (i.e., in the first half of 2019) had already heard about PrEP. During these first FGDs, participants frequently asked questions about its efficacy, how to take it, or potential side effects. In subsequent FGDs, ~88% (52/59) had heard of PrEP. These participants stated that they either knew PrEP users or had heard good things about it, such as this participant:

*Everyone said that it is a good thing, you can be more easily protected* […] *you can have better unprotected* [i.e., condomless] *sex, even without knowing the other partner, I've heard that*. – FGD10; Burkina Faso, <25 years old MSM.

Interviewees were participants in the CohMSM-PrEP study and reported that in general, they had not heard about PrEP before they started using it in the study, while others also indicated that they were informed by peers:

*A friend talked to me about PrEP, he made me understand that it's a way to protect yourself against HIV, other than condoms* […]. *The way he explained it, I found it to be something really good*. – PrEP user interview Burkina Faso.

#### 3.2.2. Attitudes toward oral PrEP

In all FGDs, participants generally had a positive attitude toward PrEP, as it could protect them against HIV. The PrEP users we interviewed indicated that they were curious, surprised, and hesitant but mainly felt positive upon learning of its existence, e.g., when enrolling in the CohMSM-PrEP study:

*It was a real joy for me, I was very happy to be able to participate* [in the study] *too because we have been waiting for something like that that could help us* – PrEP user interview, Togo.

In the FGDs and interviews, participants frequently indicated that PrEP could overcome particular difficulties that they encounter with condom use. PrEP was perceived to be better as condoms can tear and cause loss of erection, and having condoms on you can be perceived as being promiscuous. For example, one participant explained that condoms are “unnecessary luggage”:

*P1*: *I think it is a good prevention option because before having sex, you can take it without any problems, whether you know you're going out or to see your partner, you have already taken it and you don't have any luggage on you*

Moderator: *What do you mean?*

*P1: Even the fear of giving it* [the condom] *to your friends, when they open it, “Ha, so you are walking around with condoms?”. That is very inconvenient. –* FGD1; Côte d'Ivoire, <25 years old MSM.

They felt that taking PrEP would facilitate condomless sex and would therefore lead to a better sex life, as explained here:

*PrEP has advantages, positive sides, first of all because it enables the prevention of HIV infection, hmm, and secondly, it enables more fulfilling sexuality* [translated from ‘sexualité épanouie']. – FGD9; Togo, HIV positive MSM.

The FGDs with HIV-positive MSM revealed that they were particularly positive toward PrEP, as this could provide their peers with an opportunity that they did not have:

*First of all, I think* [PrEP] *is a good idea* […] *I thank the person who had the idea to create this because it will save our brothers who are behind* [i.e., do not yet have HIV]. *It will enable them to not make the same mistakes as we did*. – FGD2; Côte d'Ivoire, HIV positive MSM.

A drawback of PrEP was that it does not protect against other sexually transmitted infections (STIs). Some participants indicated that they, therefore, did not see the added value of PrEP, as this participant explains:

*As* [PrEP] *does not protect me against STIs, I don't see the effectiveness of it*. – FGD2; Côte d'Ivoire, 25+ years old MSM.

### 3.3. Suggested strategies to increase oral PrEP uptake among West African MSM

#### 3.3.1. Increase awareness and improve knowledge of HIV and HIV risk

When discussing HIV and available prevention options, FGD participants proved to be knowledgeable as a group. However, certain participants provided or reported false information (e.g., that HIV can be contracted by using public restrooms), explained that particular misconceptions exist (e.g., that HIV is invented by white people to sell condoms or to infect African MSM), or did not believe in its existence, as these participants had no firsthand or direct experience with HIV:

*P1: I like to do it without a condom. Many people talk about that, about AIDS, but I have never seen it. And I also don't know anyone who has it, so I don't think it's real*. […].

*P2: This HIV that my friend is just talking about*, […] *I have never had it, I have never dated anyone who has it* […] *I hear people talk about it, but I have never seen it; therefore, I doubt whether it is real or not*. – FGD2; Côte d'Ivoire, 25+ years old MSM.

Low self-perceived risk of HIV infection was a frequently recurring barrier mentioned in the group discussions and interviews. For example, participants explained how a negative HIV test could be perceived as a confirmation that one is not at risk of HIV infection:

*P1: As his test was negative, he refuses PrEP categorically. According to him, there is no risk*.

*P2: His result was negative despite having multiple sex partners, so he does not believe in the existence of HIV/AIDS*.

*P3: I fully agree with the ideas of* [P2]*, there are many cases like that*. – FGD6; Mali, HIV positive MSM.

FGD participants also mentioned that some MSM are not motivated to protect themselves against HIV due to religious beliefs, e.g., it may be perceived that God will decide whether or not one will contract HIV, as explained in this FGD:

*There are some who are very traditional, like, in the logic of destiny, “So it is God who says that you will grow old, you will get this, you will die one day.” So, they will not come to take PrEP*. […] “*If I don't want to take PrEP, it is God who does not make me want to take PrEP, so maybe I will not get AIDS, or if I do get AIDS, it is God who wants me to have AIDS”, so it is religious mentality like that that will hinder PrEP uptake*. – FGD11; Burkina Faso, 25+ years old MSM.

When asked how they would persuade someone to take PrEP, participants frequently stated that you must first make them understand the risks of HIV and that they are at risk of the acquisition of HIV, as explained by this participant:

*First of all, I will make him understand the consequences of the disease, explain to him that once you have contracted the disease, it is incurable, like that! Get him to understand the disease first, and then I'll explain the advantages of PrEP. –* FGD11; Burkina Faso, 25+ years old MSM.

#### 3.3.2. Improve dissemination of PrEP information to allow for informed choices

Another main barrier perceived by participants was the lack of PrEP awareness and knowledge within MSM communities. During the FGDs and interviews, it became clear that particular misconceptions about PrEP existed within these communities, which can hinder uptake, such as that PrEP also protects against other diseases (e.g., Ebola) or that it was being introduced by Europeans to trick African people, as this participant had heard:

[they said] *PrEP does not enable you to avoid getting HIV, it is a drug white people have created as a joke* – FGD3; Côte d'Ivoire, HIV-positive MSM.

Focus group discussion participants and interviewees suggested that potential users need to be better informed about PrEP to increase uptake. According to them, raising awareness should focus on eradicating doubts about its efficacy and stating the advantages and disadvantages to allow for making informed choices.

*First, we start with awareness-raising* [translated from ‘sensibilisation']*, and showing the advantages of PrEP, like the protection against HIV in the ‘letting-go', in the sense of not using a condom, and then also mention the inconveniences, like that PrEP does not protect against STIs*. – FGD2; Côte d'Ivoire, 25+ years old MSM.

When asking interviewees how they came to use PrEP in the study, the majority indicated that they were convinced by a healthcare professional or a peer who had explained PrEP to them. In the FGDs, it was considered important that such information be provided by either healthcare professionals or peers. It would be even better if they also used PrEP, as mentioned here:

*Like that, it's what* [another participant] *says, you first have to give good information and then invite some people to testify about the efficacy of PrEP. –* FGD8; Togo, 25+ years old MSM.

Interviewees and FGD participants mentioned various targeted strategies to spread information on PrEP, such as workshops, group discussions, films, and posters. Some mentioned having people testify, such as PrEP users or HIV-positive persons explaining the benefits of PrEP.

*We need to raise awareness, we can have testimonies of some sort, even if his face is covered, hidden, that could help people to understand and make it serious. There are even* [HIV positive individuals] *that could come, just to discuss the disease or “I have not taken it correctly and now I'm infected”. –* PrEP user interview, Burkina Faso.

#### 3.3.3. Implement strategies to avoid PrEP stigma

Another frequently reported barrier to PrEP uptake was the fear of being stigmatized, or being rejected by family, due to its associations with HIV, homosexuality, and promiscuity, as explained by this FGD participant:

*He* [the case vignette] *will refuse PrEP because he does not want to be seen like the others, as it is perceived to be for LGBT* […] *if someone sees him taking it, they will say, “Ah, does he not have HIV?” or like* [another participant] *said, “Is he not going to do sexual vagrancy* [i.e. sexual promiscuity, translated from ‘vagabondage sexuel']*?” if this person knows PrEP*. – FGD11; Burkina Faso, 25+ years old MSM.

Some participants suggested that PrEP information be widely disseminated, or made available to non-MSM to raise PrEP awareness and knowledge in the general population. Or, as this participant explains, it could help normalize PrEP by underscoring its ability to prevent HIV:

*It could convince them* [parents] *too, by saying that [PrEP] enables the avoidance of HIV/AIDS* [….] *in both hetero ways, like everybody avoids getting HIV/AIDS. –* FGD3; Côte d'Ivoire, HIV positive MSM.

Other strategies related to stigmatization avoidance involved hiding the pills (e.g., changing the bottle or putting PrEP into other containers). The following FGD with HIV-positive MSM demonstrates how a participant was hiding pills for his friends:

*Many MSM like us are staying with a parent. My house is like a pharmacy; many of my friends come to take their medication at my place as they are confronted with the problem of not knowing where to store it*. – FGD6; Mali, HIV positive MSM.

## 4. Discussion

The West African MSM in our study were generally positive toward oral PrEP in particular, as it could resolve difficulties they experience with condom use. According to participants, important strategies for increasing PrEP uptake in West African MSM communities are improving HIV knowledge and self-perceived risk of HIV through increased awareness-raising, increased dissemination of PrEP information, preferably via peers and healthcare professionals, and implementing strategies or tailoring PrEP delivery to avoid potential stigmatization.

Study participants considered self-perceived HIV risk to be a crucial factor for increasing PrEP uptake, in line with the literature on barriers to PrEP uptake among MSM (13, 29). Our study results suggest that awareness of HIV risk is insufficient among West African MSM communities and that misconceptions continue to exist (e.g., that HIV was created to sell condoms). The fact that some participants indicated they did not believe in HIV due to limited direct or firsthand experiences may be associated with the relatively younger age of the participants, as at least one-third of the FGD participants were below 25 years old. Young MSM may be less likely to know someone close to them living with HIV or to have lost someone to HIV (30). In a recent study among Thai MSM, it was found that young MSM are more likely to have a low self-perceived HIV risk, despite being at higher risk of HIV acquisition (31). Similarly, a study in Brazil found that young MSM were more likely to engage in high-risk sexual behavior, which the authors partially attributed to having less fear of HIV/AIDS and prevention optimism, i.e., being optimistic about HIV treatment and prevention strategies (32). Oral PrEP is a highly effective tool, and novel biomedical HIV prevention tools, such as injectable PrEP, are currently in the pipeline (33). To ensure that these are taken up in West African MSM communities, their implementation will need to be combined with strategies to increase HIV risk awareness, in particular, among young MSM.

To increase PrEP awareness, dissemination of PrEP information via peers was considered important, confirming that this is a valuable strategy (34, 35). It is estimated that ~15% of the MSM were aware of oral PrEP in 2015–2016, i.e., right before the start of the CohMSM-PrEP study (19). The results of our study reveal that PrEP awareness had already increased over the course of the study, likely via participants of the parallel CohMSM-PrEP study (24). This finding corroborates quantitative studies, indicating that oral PrEP awareness in these communities may already have increased (36, 37). Stimulating peer communication and supporting PrEP users in actively disclosing their PrEP status to other community members can be important strategies for increasing its uptake (38). Moreover, community outreach may be crucial to increasing the uptake of HIV prevention services such as PrEP and HIV testing among those not-yet-reached by local clinics or gay community organizations (23, 39, 40). Whether and to what extent such peer-supported strategies may work to increase PrEP awareness among other populations in West Africa (e.g., female sex workers) needs further study.

A frequently mentioned major barrier for oral PrEP uptake in West African MSM communities in our study was anticipated stigma due to its associations with homosexuality and HIV, which resonates with scientific literature (13). Many West African MSM are living with family (e.g., parents or female partners) (15, 23, 41), to whom they have not disclosed their sexual orientation (18, 23). Using oral PrEP may increase the risk of being identified as MSM, e.g., by pill bottles being found or being seen in or near the clinic, and subsequently lead to discrimination or social exclusion. To overcome such barriers, participants proposed to normalize PrEP by making it available to the wider public and to underscore its protective effect. As suggested elsewhere, moving away from a risk-based narrative toward a health-promotion approach may be a more convincing narrative to increase PrEP demand (38, 42, 43). Novel long-acting HIV prevention tools such as injectable PrEP may be very promising in these communities, as they are more discrete and could reduce the risk of stigmatization upon involuntary disclosure of their sexual orientation (44). However, the misconceptions and false information regarding HIV and PrEP encountered in our study, combined with the suggested fears of stigmatization, discrimination, and social rejection when taking PrEP, also point to the difficulties for HIV and community organizations to address these matters in West African MSM communities. Enabling such organizations to register officially and freely operate, abolishing laws that penalize sex between men, and providing legal protection against discrimination based on sexual orientation and HIV status continue to be highly important policy recommendations for addressing the HIV epidemic in countries such as in West Africa (22).

An important limitation is that the nature of qualitative data limits the generalizability of the findings to all West African MSM. Our participants are probably not representative of all West African MSM. For example, due to potential selection bias, focus group participants may have been more interested in taking PrEP. Furthermore, the study participants were relatively well-educated, as at least half of them had obtained or were obtaining a higher education degree. We have also chosen not to compare the collected data between countries, as we would be unable to determine whether differences are due to methodological (e.g., the local researcher) or contextual (e.g., different religions) factors. Furthermore, it should be noted that attitudes toward PrEP, knowledge, and awareness are not fixed, but may have already shifted due to the subsequent roll-out of PrEP in these countries. We recommend further research to investigate the extent of PrEP uptake among different key populations (including female sex workers) in low- and middle-income countries, to understand whether and how these barriers persist in practice. Due to the nature of FGDs and interviews, a social desirability bias cannot be excluded, e.g., more likely to find PrEP positive as it protects against HIV. Moreover, the FGDs and interviews were mostly conducted within HIV clinics, and participants were recruited via peer educators of these clinics. This could also mean that we were less able to recruit potential participants who were afraid of being associated with homosexuality or HIV.

## 5. Conclusion

In this qualitative research study, we found that West African MSM generally have positive attitudes toward PrEP but also perceived important barriers to its uptake. Based on the findings and following participants' suggestions, we recommend that the roll-out of oral PrEP and other PrEP modalities in the future should be accompanied by increased awareness-raising of HIV risk, preferably targeting young MSM. We also recommend wide dissemination of PrEP information that focuses on health promotion via peers and community organizations and tailored delivery to reduce stigmatization. Furthermore, the results point to difficulties in adequately reaching MSM with HIV prevention services due to stigma related to HIV and homosexuality. Sustained efforts to prevent discrimination and stigmatization based on HIV status or sexual orientation continue to be highly important strategies to address the HIV epidemic in West Africa.

## Data availability statement

The datasets presented in this article are not readily available because of ethical restrictions. Requests to access the datasets should be directed to treyniers@itg.be.

## Ethics statement

The studies involving human participants were reviewed and approved by the Institutional Review Board of the Institute of Tropical Medicine [1279/19], the Ethical Committee of the Ministries of Health of Burkina Faso [2019/3/025], Côte d'Ivoire [025-19/MSHP/CNESVS-kp], and Togo [21/2018/CBRS], and the University of Sciences, Techniques and Technologies of Bamako, Mali [2019/09/CE/FMPOS]. The patients/participants provided their written informed consent to participate in this study.

## Author contributions

TR, DR, AE, CA, TD, EM, BD, BS, BV, and CL were involved in conceptualizing and designing the study. TR, SB, MO, IK, LE, AE, BV, and CL operationalized and conducted data collection and analysis. TR and SB drafted the manuscript. All authors commented on subsequent versions and approved the final manuscript.
