# Supplementary material for: Strategies to improve PrEP uptake among West African men who have sex with men: a multi-country qualitative study
Source: Front Public Health. 2023 Apr 25;11:1165327. doi: 10.3389/fpubh.2023.1165327 (PMC10167043; doi:10.3389/fpubh.2023.1165327)
Supplement: Supplementary file 1 [file Data_Sheet_1.PDF]

Annex – Topic guide focus group discussions

Introduction

- Introduction des chercheurs et le projet
    - Les chercheurs
    - L'objectif du GDD
- Instructions pour ce GDD
    - Pas de mauvaises réponses
    - L'enregistrement audio
    - Pas de bruits (enregistrement audio)
    - Parler à tour de rôle (enregistrement audio)
    - Opinions différentes = discussion, mais le respect est de mise
    - Déroulement de la discussion
    - Aspects pratiques (toilettes, boissons, etc).
- Les aspects éthiques
    - Confidentialité
    - Coopération volontaire
    - Aucune obligation (à coopérer ou répondre)
- Les premières questions :
    - *Un tour de table, présentez-vous, votre prénom, + Question*

Le VIH (introduire le sujet générale)

Connaissez-vous des ***personnes qui sont infectées par le VIH*** *? Ne nous dites pas de noms, mais pensez-vous que l'infection au VIH est fréquente dans votre entourage?*

- Si besoin, demandez :
    - *Estimez-vous que le nombre d'infections a augmenté ou baissé ces dernières années? Expliquez-vous ?*
    - *Quels sont les moyens de protection que vous connaissez ?*

La PrEP (passage au sujet)

- *Dans ce groupe de discussion nous allons discuter la PrEP. Que savez-vous sur la PrEP?*

Après les réponses :

PrEP, c'est pré exposition prophylaxie, cela veut dire que c'est un traitement préventif contre le virus du VIH, pour des personnes VIH négatifs qui sont fortement exposées au risque d'infection au VIH. Ils peuvent donc prendre ces médicaments afin d'éviter une contamination. Ce sont des comprimés que vous devez prendre tous les jours, donc quotidien, ou vous pouvez prendre cela à la demande, donc avant et après un risque potentiel.

- *Qu'est ce que vous en pensez ?*

  Si besoin, pour relancer, explorez :
    - *Les aspects positifs*
    - *Les aspects négatifs*
    - *L'effet sur la santé*
    - *L'effet sur la santé sexuelle*

Les perceptions (question clé)

Dans la communauté, il y des opinions différentes sur la PrEP :

- *Qu'est ce que la communauté pense de la PrEP ?*

  Si besoin, pour relancer, demandez:
    - *Les aspects positifs*
    - *Les aspects négatifs*
    - *Demandez des exemples*

- *Qu'est-ce que la communauté pense des personnes qui prennent la PrEP (positif, négatif, exemples)*

  Si besoin, pour relancer, demandez:
    - *Les aspects positifs*
    - *Les aspects négatifs*
    - *Demandez des exemples*

Les obstacles (question clé)

Imaginez-vous un homme, Jean par exemple, qui habite à [Abidjan/Bamako/Lomé/Ouagadougou]. Jean est un homme qui a des rapports sexuels avec d'autres hommes, mais il n'a pas de partenaire stable. Il lui arrive parfois de ne pas utiliser de préservatif, ou que le préservatif casse. Il est clair que Jean court un risque d'infection au VIH élevé. Lors de sa dernière visite au centre, il a fait un test de VIH, qui s'est avéré négatif. On lui a proposé la PrEP, mais il a refusé.

- *Selon vous, quelles sont les raisons pour lesquelles Jean refuse la PrEP*

Si besoin, pour relancer, explorez:

- Des exemples
- Des expériences

- *Laquelle est, selon vous, la raison principale ?*

Si besoin, demandez les différences entre les raisons.

Les stratégies proposés pour améliorer l'adoption de la PrEP (question clé)

Qu'est-ce que l'on peut faire pour que Jean accepte la PrEP ?

PrEP & Préservatifs (questions finales)

Dès qu'il a commencé avec la PrEP, Jean commence a utiliser moins de préservatifs. Qu'en pensez-vous de cela ?

Explorez :

- La place des préservatifs dans la prévention
- La place des préservatifs à côté de la PrEP

Fin
